# Re-emergence of foot-and-mouth disease in the Republic of Korea caused by the O/ME-SA/Ind-2001e lineage

**DOI:** 10.3389/fvets.2024.1378769

**Published:** 2024-04-10

**Authors:** Soyoon Ryoo, Hyeonjeong Kang, Da-Rae Lim, Jae-Myung Kim, Youngwoo Won, Ji Ye Kim, Donald P. King, Antonello Di Nardo, Sang-Ho Cha

**Affiliations:** ^1^Foot-and-Mouth Disease Diagnostic Division, Animal and Plant Quarantine Agency, Gimcheon, Republic of Korea; ^2^Chungcheongbuk-do Livestock and Veterinary Service, Cheongju, Republic of Korea; ^3^The Pirbright Institute, Surrey, United Kingdom

**Keywords:** foot-and-mouth disease virus, outbreak, ME-SA, Ind-2001e, Korea, vaccination

## Abstract

The O/ME-SA/Ind-2001e foot-and-mouth disease virus (FMDV) lineage is a pandemic strain that has recently become dominant within East and Southeast Asia. During May 2023, this viral lineage spread to the Republic of Korea, where 11 outbreaks were detected on cattle and goat farms located in Cheongju and Jeungpyeong. Infected animals displayed typical FMD signs including vesicular lesions with drooling and anorexia. Molecular diagnostic testing and genetic analysis (VP1 sequencing) showed that the causative FMDVs belonged to the O/ME-SA/Ind-2001e lineage and shared the closest nucleotide identity (97.95–99.21%) to viruses that have been collected from Mongolia and South-East Asian countries. Phylogenetic analyses showed that these sequences were distinct to those collected from the previous Korean O/ME-SA/Ind-2001e lineage outbreaks in 2019, demonstrating that these cases are due to a new incursion of the virus into the country. Prompt implementation of emergency vaccination using antigenically matched serotype O vaccines (r1 value: 0.74–0.93), together with intensive active surveillance on farms surrounding the infected premises has successfully prevented further spread of FMD. These recent FMD outbreaks reinforce the importance of research to understand the risks associated with transboundary pathways in the region, in order to reduce the possibility of a further reintroduction of FMD into the Republic of Korea.

## 1 Introduction

Foot-and-mouth disease (FMD) is one of the most important animal diseases. FMD is highly contagious to cloven-hoofed animals and causes severe economic losses due to its devastating impact on trade and reduced animal productivity (1). The causative agent is the foot-and-mouth disease virus (FMDV), which belongs to the genus *Aphthovirus* of the family *Picornaviridae*. FMDV is divided into seven genetically and immunologically distinct serotypes [O, A, Asia 1, C, Southern African Territories (SAT) 1, SAT 2 and SAT 3] including multiple topotypes and genetic lineages (2, 3). FMDV strains circulating throughout the world have been geographically divided into seven regional virus pools (1, 4). Serotype O, A, and Asia 1 viruses circulate in the Asian pools (Pools 1, 2, and 3), which pose threats to livestock industries in countries such as the Republic of Korea.

Serotype O is the most widely distributed of the seven FMDV serotypes. In Southeast Asia and East Asia (Pool 1), serotype O viruses are responsible for over 80% of the officially reported outbreaks. Type O viruses belonging to the SEA (O/SEA/Mya-98), ME-SA (O/ME-SA/PanAsia, O/ME-SA/Ind-2001) and CATHAY topotypes are endemic in this region. The O/ME-SA/Ind-2001 lineage has been classified into five sublineages, namely, a, b, c, d, and e. Since 2020, there has been an upward trend in the dominance of the O/Ind-2001e sublineage in Southeast Asian countries (5).

The Republic of Korea was free from FMD from 1934 until 2000. However, since 2000, FMD has been introduced into the country on multiple occasions. FMD outbreaks in 2010 caused significant economic damage estimated at more than US$ 3.6 billion (6). As a consequence, a national program that ensured that all FMD-susceptible animals were vaccinated began in early 2011. However, despite this strong nationwide vaccination policies, FMD recurred every year from 2014 to 2019, recording a total of 11 incursions since 2000 caused by both FMD type O and A viruses (7, 8). The genotypes of FMDV that have been detected in the Republic of Korea are O/ME-SA/PanAsia, O/ME-SA/Ind-2001e, O/SEA/Mya-98 and A/ASIA/Sea-97. During 9 days in May 2023, new FMD clinical cases were reported on ten cattle and one goat farms located in two regions, Cheongju and Jeungpyeong of Chungbuk. The FMD-reported animals showed typical clinical signs and lesions, such as anorexia, vesicular fluids, erosion, and drooling. In this study, genetic characterization of the causative virus and vaccine matching test using a virus isolated from clinical samples were conducted to investigate (i) the epidemiological relationship with contemporary FMD viruses circulating in neighboring regions, and (ii) the antigenic relatedness of commercial vaccine strains used for domestic compulsory vaccination. These data help to understand the risk pathways by which FMDVs enter the Republic of Korea and provide data to support the national vaccination control strategy.

## 2 Materials and methods

### 2.1 Clinical samples and virus isolation

A total of 54 clinical samples (epithelium, saliva, and tissue) were collected from cattle and goats exhibiting clinical signs of FMD in FMD-affected farms located in one province of Republic of Korea. The samples were transported to the laboratory in transport media (BD Diagnostics). Tissue samples were processed by homogenization of 50 mg of tissue in DMEM media (without Fetal bovine serum) using a bead-based homogenizer. The LFBK-ανβ_6_ cell lines (supplied by the Plum Island Animal Disease Center, United States) were cultured using DMEM media (Gibco) supplemented 1% antibiotic-antimycotic (Gibco) and 10% fetal bovine serum (Atlas) for viral isolation (9). Inoculated cells were harvested when 90% of cytopathic effect (CPE) were observed, the cells and supernatant were then frozen and thawed three times, harvested and stored at −80°C until use in aliquot. If no CPE was observed, the cells were frozen and thawed, and the supernatant was used to be inoculated onto new fresh cells, which were examined for CPE for another 48 h.

### 2.2 Lateral flow strip test for rapid detection of FMDV serotypes A, O and Asia1

Clinical samples collected from 11 farms with suspect FMD cases were tested using the VDRG^®^ FMDV 3Diff/PAN Ag Rapid Kit (Median Diagnostics, Korea). Fresh vesicular fluid was collected using a syringe and added to a test tube containing 250 μL of sample dilution buffer. Tissue extracted fluid was added to 1 mL of sample dilution buffer to the tissue extraction vial. Then, after adding the tissue sample to the extraction vial, cut the tissue into pieces using scissors and grind it using the pestle and sand included in the kit. Remove the VDRG^®^ FMDV 3Diff/PAN Rapid Test Device (LFD) from the foil pouch, place it on a flat surface, and slowly add 100 μL of the treated sample to the “S1” and “S2” positions. The results were read after exactly 15 min. Specimen inlet S1 shows the three test lines O (Type O), A (Type A), and AS (Type Asia1) and the control line (C) position, while specimen inlet S2 display test line PAN (FMDV common) and the control line (C) position.

### 2.3 RNA extraction and real-time RT-PCR

Viral RNA was extracted from clinical samples and cell-isolated viruses using Nextractor^®^ NX-48 s viral NA kit (Genolution, South Korea) according to the manufacturer’s instructions. Extracted samples were tested for the presence of FMDV RNA using primers and probes directed toward the conserved 3D gene (10). PowerChek™ FMDV Multiplex Real-time PCR kit (3D/IRES) (Kogenebiotech, South Korea) was used for the amplification of 3D gene, according to the manufacturer’s instruction. The VDx FMDV O genotyping qRT-PCR set (Median Diagnostics, Korea) was used to differentiate lineages (O/Ind-2001, O/PanAsia, O/Mya-98, and O/CATHAY) by VP1 gene amplification according to the manufacturer’s protocol.

### 2.4 Sequencing and phylogenetic analysis

The VP1 coding region was amplified using universal primers as detailed in (11). DNA Sanger sequencing was performed for the purified PCR product using Big Dye Terminator v3.1 Cycle sequencing kit (Thermofisher, United States) and run on an ABI 3730 DNA analyzer (Thermofisher, United States). FMDV VP1 coding sequences (*n* = 267) were retrieved from both the Animal and Plant Quarantine Agency (APQA) database and WRLFMD database of FMDV sequences,^1^ which also includes FMDV genomes deposited in GenBank. These sequences were aligned using CLC workbench 23 (Qiagen, United States) and the homology of the nucleotide (NT) or amino acid (AA) sequences among the viral isolates was determined. VP1 coding sequences were subjected to phylogenetic trees including those of Korean field FMDV viruses (*n* = 11) isolated from 2017 to 2023, field isolates collected from neighboring countries from 2016 to 2022 (*n* = 221), and also by including FMDV prototype strains (*n* = 35). The phylogenetic tree was constructed using the Maximum Likelihood method based on the GTR (generalized time reversible) model (12), bootstrapping over 1,000 replicates using MEGA software version X (13).

Bayesian analyses were performed using BEAST version 1.10.4 (14). The evolution of FMDV was modelled by parameterizing the process of nucleotide substitution model using gamma-discretized among-site rate variation GTR model (GTR+ Γ_4_). Time to most recent common ancestor (TMRCA) for lineages were estimated in BEAST v1.10.4 employing a log-normal distributed relaxed molecular clock (15) and the non-parametric Skygrid coalescent population prior with 100 transition points (16). The posterior trajectories were acquired by running a Markov chain Monte Carlo (MCMC) for 200 million iterations, while recovering samples at intervals of 20,000 states. Mixing and convergence of the MCMC chain was assessed using Tracer 1.7.3 (17), ensuring sufficient sampling was achieved. The Maximum Clade Credibility (MCC) tree was generated by TreeAnnotator from 9,000 posterior trees, following the elimination of the initial 10% of sampled trees as burnin.

### 2.5 Two-dimensional virus neutralization test for strain differentiation

To identify a suitable FMD vaccine, FMD viruses isolated in South Korea were sent to the World Reference Laboratory for FMD (WRLFMD, Pirbright, United Kingdom). Virus neutralization test (VNT) was performed according to the WOAH Manual of Diagnostic Tests and Vaccines for Terrestrial Animals (18) on IB-RS2 cells. VNT was performed to determine the serological relationships (*r*-value) between field isolates recovered during the May 2023 Korean outbreak and reference FMDV serotype O vaccine strains of three commercial vaccines used for compulsory vaccination in the Republic of Korea.

## 3 Results

### 3.1 Sample collection and diagnosis

**Figure 1:**
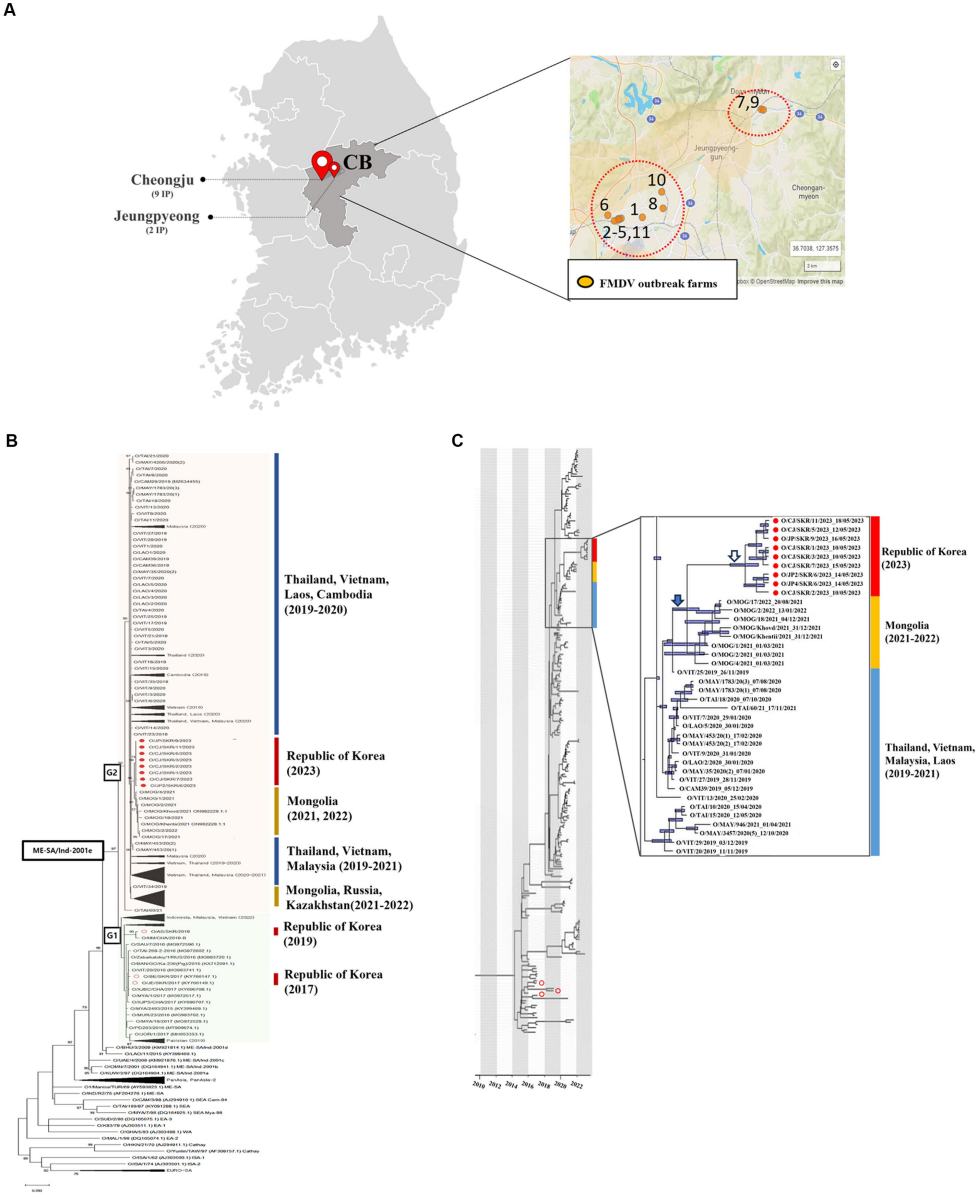
**(A)** Location and number of farms infected. **(B)** Maximum likelihood tree showing the FMDV VP1 coding sequence of the O/ME-SA/Ind-2001e lineage that caused an outbreak in the Republic of Korea in 2023 (filled red circles) and 2017 and 2019 (empty red circles) and its relationship to other recent FMD type O viruses circulating in the surrounding region. The percentages of replicate trees in which the associated taxa clustered together in the bootstrap test (1,000 replicates) are shown next to the branches. **(C)** The MCC tree displays the isolates identified during the 2023 outbreak in the Republic of Korea (filled red circles) and the 2017 and 2019 outbreaks (empty red circles). It also indicates their correlation with other O/ME-SA/Ind-2001e lineages prevalent in the neighboring countries of the Republic of Korea. 95% High Posterior Density (HPD) intervals for the timing of node ancestry are represented by slate blue bars in the MCC tree. The enlarged box contains isolates from neighboring countries that have an MRCA with the FMDV isolated in the Republic of Korea in 2023. Also included in this box is the MRCA of FMDV isolated in the Republic of Korea in 2023 (empty arrow), the MRCA of FMDV isolated in the Republic of Korea in 2023, and FMDV isolated in Mongolia in 2021-2022 (filled arrow).

Between the 10th and the 18th of May 2023, a total of 11 cases of FMD were confirmed in two regions (Cheongju and Jeungpyeong) of Chungbuk in the Republic of Korea (Figure 1A). These were identified following four reports of FMD suspicion and screening animals. All livestock on the 11 farms were clinically examined, and samples (both epithelium and saliva) were collected from animals with suspected FMD clinical signs. Samples such as epithelial fluid were tested for FMD using LFD which confirmed the FMDV positive (serotype O) status of samples from 8 farms. A total of 36 samples were collected from suspected animals on 11 farms and subjected to 3D rRT-PCR, which detected the FMDV in 40 samples from all 11 farms. These samples had Ct values ranging from 16.08 to 38.59. Of the 40 FMDV positive samples, samples from eight farms (*n* = 31) were sequenced for the VP1 coding gene, and samples from three farms (*n* = 9) where the VP1 coding gene was not amplified were identified as O/ME-SA/Ind-2001 lineage by genotyping rRT-PCR (Table 1). Twenty FMDV positive samples from 11 farms were inoculated into cells, and nine FMDVs from 8 farms were isolated.

**Table 1:** Details of the clinical samples obtained from the 11 FMD-positive farms reported during May 2023 in the Republic of Korea.

Farm No.	Category	Date	City	Species	No. of samples	LFD (Pan/Type)	Clinical sign	Sample type	rRT-PCR (3D/IRES)	Genotype
1	Report	10 May	CJ	Cattle	12	+/O	Vesicle	epithelium, saliva	Positive	O/ME-SA/Ind-2001e
2	Report	10 May	CJ	Cattle	2	+/O	Vesicle	tissue, saliva	Positive	O/ME-SA/Ind-2001e
3	Surveillance	10 May	CJ	Cattle	2	+/O	Salivation	tissue, saliva	Positive	O/ME-SA/Ind-2001e
4	Surveillance	11 May	CJ	Cattle	1	−/−	Salivation, erosion ulcer	tissue, saliva	Positive	O/ME-SA/Ind-2001 (by rRT-PCR)
5	Surveillance	12 May	CJ	Cattle	3	+/O	Salivation, ulcer	tissue, saliva	Positive	O/ME-SA/Ind-2001e
6	Report	14 May	JP	Cattle	4	+/O	ulcer	epithelium, tissue	Positive	O/ME-SA/Ind-2001e
7	Report	15 May	CJ	Cattle	3	+/O	Salivation, ulcer	tissue	Positive	O/ME-SA/Ind-2001e
8	Surveillance	16 May	CJ	Cattle	3	−/−	Salivation	saliva	Positive	O/ME-SA/Ind-2001 (by rRT-PCR)
9	Surveillance	16 Ma	JP	Cattle	4	+/O	Salivation	saliva	Positive	O/ME-SA/Ind-2001e
10	Surveillance	16 Ma	CJ	Goat	1	−/−	Salivation	saliva	Positive	O/ME-SA/Ind-2001 (by rRT-PCR)
11	Surveillance	18 Ma	CJ	Cattle	3	+/O	Salivation, ulcer	epithelium, saliva	Positive	O/ME-SA/Ind-2001e

*CJ, Cheongju; JP, Jeungpyeong.**Samples from three farms (No. 4, 8, 10) tested negative for FMDV in LFD had Ct values above 24 in 3D rRT-PCR.

### 3.2 Sequence homology and phylogenetic tree analysis

Sequencing the VP1 coding gene (1D) using samples that were positive using the FMDV 3D real-time RT-PCR confirmed the O/ME-SA/Ind-2001e lineage in eight farms. However, it was not possible to amplify the VP1 region for samples collected from the remaining three farms. The VP1 sequences analyzed in this study have been submitted to GenBank under accession numbers PP156917-PP156919.

For the 2023 Korean isolates, the nucleotide identities of the VP1 coding region varied from 99.68 to 100% and all the sequences were 100% identical to each other at the amino acid level. Based on the virus isolated from the first farm in 2023, the VP1 coding sequence was 95.73 and 94.79% identical to viruses isolated in 2017 (Boeun) and 2019 (Anseong) from the Republic of Korea, respectively, whilst percentage of identity values between 97.95 and 99.2% were estimated from FMDV isolates collected from neighboring Asian countries during 2019 and 2022 (Table 2).

**Table 2:** VP1 coding sequences of O/ME-SA/Ind-2001e FMDVs generated in this study.

Name	Collected date	Country	Host	Id (%)	Accession no.	References
CJ/SKR/1/2023	May-23	Republic of Korea	Cattle	Ref	PP156917	This study
CJ/SKR/2/2023	May-23	Republic of Korea	Cattle	100	–	This study
CJ/SKR/3/2023	May-23	Republic of Korea	Cattle	100	–	This study
CJ/SKR/5/2023	May-23	Republic of Korea	Cattle	100	–	This study
CJ/SKR/7/2023	May-23	Republic of Korea	Cattle	99.84	PP1569179	This study
JP2/SKR/6/2023	May-23	Republic of Korea	Cattle	99.68	PP1569178	This study
JP/SKR/9/2023	May-23	Republic of Korea	Cattle	100	–	This study
CJ/SKR/11/2023	May-23	Republic of Korea	Cattle	100	–	This study
JE/SKR/2017	February-17	Republic of Korea	Cattle	96.37	KY766149.1	–
BE/SKR/2017	February-17	Republic of Korea	Cattle	95.73	KY766147.1	–
AS/SKR/2019	January-19	Republic of Korea	Cattle	94.79	n/a	APQA
MOG/1/2021	March-21	Mongolia	n/a	99.21	n/a	WRLFMD
MOG/4/2021	March-21	Mongolia	n/a	99.05	n/a	WRLFMD
LAO/3/2020	January-20	Lao PDR	Cattle	98.89	n/a	WRLFMD
TAI/4/2020	May-20	Thailand	Cattle	98.89	n/a	WRLFMD
VIT/7/2020	January-20	Vietnam	Pig	98.89	n/a	WRLFMD
VIT/28/2019	November-19	Vietnam	Water buffalo	98.89	n/a	WRLFMD
MAY/35/2020 (2)	January-20	Malaysia	Cattle	98.89	n/a	WRLFMD
CAM36/2019	October-19	Cambodia	Cattle	98.89	n/a	APQA
CAM39/2019	December-19	Cambodia	Cattle	98.89	n/a	APQA
LAO1/2020	January-20	Lao PDR	Cattle	98.89	n/a	APQA
VIT1/2020	January-20	Vietnam	Cattle	98.89	n/a	APQA
MOG/2/2021	March-21	Mongolia		98.74	n/a	WRLFMD
MOG/Khentii/2021	December-21	Mongolia	Cattle	98.74	ON982228.1	Viktor et al. (27)
CAM30/2019	January-19	Cambodia	Cattle	98.74	MZ634456.1	Ryoo et al. (28)
MAY/453/20 (2)	February-20	Malaysia	–	98.74	n/a	WRLFMD
LAO/1/2020	–	Lao PDR	–	98.74	n/a	WRLFMD
MAY/127/2020	–	Malaysia	–	98.74	n/a	WRLFMD
MAY/9/2020	–	Malaysia	–	98.74	n/a	WRLFMD
VIT/23/2019	–	Vietnam	–	98.74	n/a	WRLFMD
VIT/22/2019	–	Vietnam	–	98.74	n/a	WRLFMD
VIT/21/2019	–	Vietnam	–	98.74	n/a	WRLFMD
MOG/17/2021	–	Mongolia	–	98.74	n/a	WRLFMD
MOG/2/2022	–	Mongolia	–	98.74	n/a	WRLFMD
CAM29/2019	January-19	Cambodia	Cattle	98.54	MZ634455.1	Ryoo et al. (28)
MOG/Khovd/2021	December-21	Mongolia	Cattle	98.26	ON9822298.1	Viktor et al. (27)
CAM22/2019	January-19	Cambodia	Cattle	98.10	MZ634454.1	Ryoo et al. (28)
MOG/Sukhbaatar/2021	December-21	Mongolia	Cattle	97.95	ON982230.1	Viktor et al. (27)
Orenburg/RUS/2021	December-21	Russia	Cattle	97.16	ON982231.1	Viktor et al. (27)
KAZ/2022	January-22	Kazahstan	Cattle	96.37	ON982227.1	Viktor et al. (27)

Includes FMDV VP1 coding sequences from neighboring East and Southeast Asian countries with more than 95% sequence identity to the CJ/SKR/1/2023 isolate from Chungbuk province.

A phylogenetic analysis was performed using FMDV sequences (*n* = 267) encoding the VP1 region (alignment of 633 nt in length) of the O/ME-SA/Ind-2001e lineage collected from East and Southeast Asia regions, including the ones from the Republic of Korea. The resulting topology clustered the O/ME-SA/Ind-2001e lineage into two main groups, here named as G1 and G2 (Figure 1). G1 included field viruses isolated from FMDV endemic countries in Pool 2, such as India, Bangladesh, and Bhutan, and Pool 1 such as Myanmar, Thailand, Vietnam, Russia, and China collected between 2016 and 2019. In addition, this group also contained field viruses isolated from Indonesia in 2022 as well as those collected from the Republic of Korea in 2017 and 2019. The G2 phylogenetic cluster included field viruses from Southeast Asia region (Vietnam, Cambodia, Laos, Thailand, Malaysia) and some more recent FMDV isolates (2019–2022) from outbreaks reported in Mongolia, Russia, and Kazakhstan. Thus, FMD viruses causing the 2023 outbreak in the Republic of Korea were assigned to G2 and shared a common ancestor with the FMDV isolates from Mongolia in 2021, with its MRCA dated Aug-2020 (95% BCI March 2020 to January 2021).

### 3.3 Vaccine matching

The antigenic relationship between a single field isolate (O/JP2/SKR/6/2023) and the serotype O FMDV vaccine strains (O3039, O Manisa, O1 Campos) used in the Republic of Korea was evaluated in 2D-VNT using bovine vaccine serum raised against the vaccine strains. The r1 values between the field viruses and vaccine strains were all higher than the 0.3 cut-off for expected protection (Table 3).

**Table 3:** Antigenic relationship (r_1_ values) of FMD viruses from the Republic of Korea against the currently used FMDV vaccine strains, as determined by 2D-VNT assay.

	Vaccine strain		
Field isolates	O3039	O Manisa	O1 Campos
O/JP/SKR/2/2023	0.93	0.74	0.88
O/SKR/1/2019	0.62	0.52	ND*
O/SKR/1/2017	0.48	0.43	ND
O/SKR/2/2017	0.59	0.38	ND

Data for the FMDV isolate collected during the 2023 outbreak is compared to previous outbreaks in 2017 and 2019 against with the currently used FMDV vaccine strains, as determined by 2D-VNT assay (*ND: not done, cattle not vaccinated with O campos in 2017 and 2019).

## 4 Discussion

There had been no FMD outbreaks detected in livestock of the Republic of Korea after the 11th FMD incursion reported in Anseong during January 2019. In the recent outbreaks of May 2023, a lateral flow device was used to rapidly detect FMDV in samples collected from animals exhibiting FMD clinical signs. Further testing using 3D and VP1 real-time RT-PCR confirmed FMD and serotype of FMDV, and sequencing of VP1 coding region revealed that the FMDV strain introduced into the Republic of Korea belonged to O/ME-SA/Ind-2001e sub-lineage. A national livestock standstill with culling and disinfection of the infected farms was implemented. Furthermore, emergency vaccination was conducted for all FMD-susceptible animals nationwide. These measures have been successful since no further FMD-suspected case has been identified since the last case reported the 18th of May 2023.

The thorough national epidemiological investigation suggested FMDV introduction possibly from neighboring countries via illegal animal products, based on the following assumptions: (i) for the past 4 years after the last FMD outbreak of 2019, a strict FMD reporting system and a post-vaccination monitoring (PVM) program involving 1.2 millions of animals have been implemented for all FMDV susceptible livestock in the country; these systems have not detected any FMD-suspected cases nor evidence of FMDV infection from non-negative NSP-positive animals identified during surveillance; finding which were confirmed by real-time RT-PCR testing of clinical samples of the NSP-non-negative animals as well as other animals from these farms; (ii) FMDV and 5 antibody test using antisera (*n* = 8,681) of wild animals (wild boar, water deer, roe deer, etc.) during the last 5 years from 2019 to June 2023 showed no FMDV infection in wild animal populations of the Republic of Korea (data not shown); (iii) the FMDV isolates genetically characterized from the 2023 outbreak belonged to the O/ME-SA/Ind-2001e, clustering in a phylogenetic group that was distinct from those viruses of the same lineage that caused the outbreaks reported in 2017 and 2019, with 5–6% of nucleotide difference in the FMDV sequences of the VP1 coding region. From the phylogenetic tree, the 2023 FMD viruses were closely related to field viruses (O/ME-SA/Ind-2001e) isolated between 2022 and 2021 from Mongolia and other Southeast Asian countries (Laos, Vietnam, and Cambodia) with ~1% of nucleotide sequence difference, clustering within the same group. This FMDV lineage, is the predominant strain circulating within FMD endemic Pool 1, as was reported during 2020-2023 (19, 20); (iv) Inspections of illegal livestock products found about 60 cases/day in the luggage of travelers visiting neighboring countries and about 70 cases/day in overseas express cargo. In addition, inspections of foreign grocery stores in cities and counties adjacent to the FMD outbreak areas confirmed the illegal sale of animal products.

It was confirmed by vaccine-matching tests that type O FMDV strains of the commercial vaccines used for compulsory systemic vaccination policy in the Republic of Korea were well matched (r1 value >0.3) against the FMD field isolates (Table 3), including those FMDV isolates causing outbreaks in 2017 and 2019. Therefore, it is believed that the emergency vaccination should be highly effective to contain the FMD outbreaks within limited areas (two regions in a province) for a short period of time (9 days), as demonstrated in swift control of FMD outbreaks during the past two outbreaks (2017 and 2019) reported in the Republic of Korea. The effect of emergency vaccination as one of major control measures in emergency FMD outbreaks (18, 21) was already proven by previous studies (22).

During the nationwide vaccination policy, post-vaccination monitoring (PVM) showed that over 90% of FMD-susceptible livestock had seroconverted (23), satisfying 80% population herd immunity on average, which was also confirmed in the recent PVM carried out between January and June of 2023 (data not shown). Nevertheless, the likely cause of FMDV introduction and transmission within a systematically vaccinated population might be occur if the levels of protective immunity are low as observed on some farms. As shown in a previous study (23), around 0.3–0.79% of farms were found to have less than 80% of herd immunity, despite the effective vaccination policy, with presence of livestock farms having low herd immunity was still observed during the recent PVM carried out in 2023. In general, the level of herd immunity depends on the reproduction ratio (R_0_) as well as effectiveness of vaccination. 75–80% coverage of population herd immunity is usually referenced as the level of immunity required to block the spread of FMDV (24, 25), when R_0_ of an FMD outbreak is estimated to be below 5 and vaccination is 100% effective (26). SP/O ELISA was conducted in all the 11 farms reported of being infected by FMD in Chungbuk Province, where results revealed a positive rate between 24 and 75% for 7 farms (data not shown). Understanding these results is important because even if the vaccine is well matched and high potency, any non-compliance with vaccination procedures prescribed by manufacturer or the Republic of Korea Government guidelines may lead to immunity gaps on farms. Testing of samples from the remaining four FMD-positive farms revealed over 80% SP/O ELISA positive rate, which might be results of anamnestic responses induced by viral infection following vaccination.

After a period of 4 years where no FMD outbreaks have been detected, this report highlights the reintroduction of the O/ME-SA/Ind-2001e FMDV lineage into the Republic of Korea during May of 2023. The active circulation of this lineage in neighboring countries suggest a transboundary origin for these cases, which was further supported by the close genetic relatedness between the field viruses of the same FMDV lineage isolated from other East Asian countries between 2021 and 2022. It was noted that the outbreaks occurred on farms with a low level of serotype O-specific antibodies despite the systemic compulsory vaccination policy. These findings reinforce the importance of maintaining the protective immune status against FMDV to prevent the occurrence of FMD in geographic situations where FMDV may be introduced.

## Data availability statement

The datasets presented in this study can be found in online repositories. The names of the repository/repositories and accession number (s) can be found in the article/supplementary material.

## Ethics statement

Ethical approval was not required for the study involving animals in accordance with the local legislation and institutional requirements because No ethical approval was required for this study as sample collection was performed using standard diagnostic procedures with no harm to the animals. Written informed consent was obtained from the owners for the participation of their animals in this study.

## Author contributions

SR: Conceptualization, Data curation, Formal analysis, Visualization, Writing – original draft, Writing – review & editing. HK: Data curation, Formal analysis, Visualization, Writing – original draft, Writing – review & editing. D-RL: Data curation, Formal analysis, Visualization, Writing – original draft. J-MK: Project administration, Writing – review & editing. YW: Investigation, Writing – review & editing. JK: Investigation, Writing – review & editing. DK: Data curation, Formal analysis, Writing – review & editing. AN: Writing – review & editing, Data curation, Formal analysis. S-HC: Conceptualization, Data curation, Formal analysis, Supervision, Writing – original draft, Writing – review & editing.

## Footnotes

^1^ www.fmdbase.org
